# Identifying a group of factors predicting cognitive impairment among older adults

**DOI:** 10.1371/journal.pone.0301979

**Published:** 2024-04-11

**Authors:** Longgang Zhao, Yuan Wang, Eric Mishio Bawa, Zichun Meng, Jingkai Wei, Sarah Newman-Norlund, Tushar Trivedi, Hatice Hasturk, Roger D. Newman-Norlund, Julius Fridriksson, Anwar T. Merchant

**Affiliations:** 1 Department of Epidemiology and Biostatistics, Arnold School of Public Health, University of South Carolina, Columbia, South Carolina, United States of America; 2 Communication Sciences and Disorders, Arnold School of Public Health, University of South Carolina, Columbia, South Carolina, United States of America; 3 Regional Medical Center Primary Care Stroke, Orangeburg, SC, United States of America; 4 Center for Clinical and Translational Research, Forsyth Institute, Boston, MA, United States of America; Florida State University, UNITED STATES

## Abstract

**Background:**

Cognitive impairment has multiple risk factors spanning several domains, but few studies have evaluated risk factor clusters. We aimed to identify naturally occurring clusters of risk factors of poor cognition among middle-aged and older adults and evaluate associations between measures of cognition and these risk factor clusters.

**Methods:**

We used data from the National Health and Nutrition Examination Survey (NHANES) III (training dataset, n = 4074) and the NHANES 2011–2014 (validation dataset, n = 2510). Risk factors were selected based on the literature. We used both traditional logistic models and support vector machine methods to construct a composite score of risk factor clusters. We evaluated associations between the risk score and cognitive performance using the logistic model by estimating odds ratios (OR) and 95% confidence intervals (CI).

**Results:**

Using the training dataset, we developed a composite risk score that predicted undiagnosed cognitive decline based on ten selected predictive risk factors including age, waist circumference, healthy eating index, race, education, income, physical activity, diabetes, hypercholesterolemia, and annual visit to dentist. The risk score was significantly associated with poor cognitive performance both in the training dataset (OR _Tertile 3 verse tertile 1_ = 8.15, 95% CI: 5.36–12.4) and validation dataset (OR _Tertile 3 verse tertile 1_ = 4.31, 95% CI: 2.62–7.08). The area under the receiver operating characteristics curve for the predictive model was 0.74 and 0.77 for crude model and model adjusted for age, sex, and race.

**Conclusion:**

The model based on selected risk factors may be used to identify high risk individuals with cognitive impairment.

## Introduction

Between 29% and 76% of patients with dementia may remain undiagnosed [1–3] even though the Centers for Medicare and Medicaid Services recommends the assessment of impaired cognition for older individuals at annual wellness visits. As routine screening for impaired cognition is not recommended, evaluation or diagnosis of impaired cognition results from complaints by the individual or caregiver or provider suspicion [4].

Increasing age and the ε4 allele of the apolipoprotein E gene are the strongest predictors of impaired cognition [5], but several other factors are correlated with impaired cognition including blood pressure, diabetes, smoking, alcohol use, diet, physical activity, serum cholesterol [6, 7], and oral health status [8]. With the exception of age and the ε4 allele of the apolipoprotein E gene, these factors are potentially modifiable to prevent or delay cognitive decline. Blood pressure, blood sugar, serum cholesterol smoking, alcohol use, diet, and physical activity, were part of the Brain Care Score which predicted incident dementia, suggesting that improving the Brain Risk Score could reduce dementia risk [9]. While information on these risk factors is often present in medical records, a formal way of including them in the assessment of impaired cognition is challenging. We included information on oral health status in addition to other risk factors, including those mentioned above, because oral health status is correlated with poor cognition but infrequently included in risk scores. Moreover, this information is typically present in electronic health records and was available in the dataset that we were planning to use.

The goal of this study was to create and validate a score predicting the prevalence of impaired cognition in older adults from information on known risk factors using statistical and machine learning methods. A validated algorithm embedded into electronic health records could use pertinent information available in health records to calculate the likelihood of impaired cognition of their patient using all available data and cue the health care provider. Formal screening of individuals identified following this impaired cognition score could potentially increase the positive predictive value of existing screening tools such as the Mini-Mental State Examination (MMSE).

## Methods

### Data source

We used a subset of cross-sectional data from the Third National Health and Nutrition Examination Survey (NHANES III) which had measures of cognition for participants 40 years and older [10] and its commonly known risk factors as the data source to develop a impaired cognition score predicting impaired cognition. We called this the training dataset. The validation dataset consisted of cross-sectional information from participants of continuous NHANES surveys 2011 through 2014 who were 60 years and older with data on cognitive assessment and covariates [11]. This validation dataset was used to confirm the ability of the impaired cognition score developed in the training dataset to predict impaired cognition, and estimate its sensitivity and specificity.

There were 7,869 participants in the training dataset (NHANES III) and 19,931 participants in the validation dataset (NHANES 2011–2014). In the training dataset, 3795 participants were excluded because of missing values in covariates or cognition variables. Similarly, 16,259 participants in the validation dataset were excluded for no valid measurement for cognition and 962 for missing values in covariates. After exclusions, 4074 and 2510 participants remained in the training and validation datasets respectively (**S1 Fig in S1 Appendix**). NHANES III participants consented to participate in the study voluntarily. The data used in these analyses was deidentified and classified as exempt from review by the Institutional Review Board of the University of South Carolina.

### Exposure assessment

#### Training dataset

Predictive factors consisted of **sociodemographic factors** which included age, sex (male and female), race (white, black, and other), educational level (<12 years, ≥12 years completed education), poverty income ratio (PIR) divided into three groups (≤1.3, 1.3<PIR≤3.5, >3.5) (a value <1 indicates that household income is below poverty level, a value of 1.3 means that household income is 30% above poverty level and so on. The higher this number the wealthier the household), **lifestyle factors** consisting of smoking status (non-smoker, ever smoker, and current smoker), drinking status (non-drinker and drinker), healthy eating index (HEI) (higher number indicating healthier diet), **health related factors** including, body mass index (BMI) (normal: ≤24.9 kg/m^2^; overweight: 25 to ≤29.9 kg/m^2^, and obese: ≥30 kg/m^2^), waist circumference (in cm), physical activity (derived based on a structured physical activity questionnaire, and further classified as three groups: sedentary, moderate, vigorous), systolic blood pressure (SBP), diastolic blood pressure (DBP), white blood cell count (WBC), C-reactive protein (CRP), total cholesterol, high density lipoprotein cholesterol (HDL), triglycerides, **disease prevalence** including diabetes, hypertension, cardiovascular diseases, cancer, and depression, **oral health** information consisting of periodontal disease (none or mild versus moderate or severe) [12, 13], and annual dentist visits, and **social connectedness** measures consisting of frequency of talking to friends and family, visiting friends and family, attending church services and club meetings. Details about these variables are available at the CDC website [11, 12].

#### Validation dataset

Covariates measured included age in years, sex (male and female), race (white, black, and other), education ((<12 years, ≥12 years completed education), PIR (≤1.3, 1.3<PIR≤3.5, >3.5), smoking (non-smoker, ever smoker, and current smoker), drinking status (non-drinker and drinker), HEI score, BMI (normal: ≤24.9 kg/m^2^; overweight: 25 to ≤29.9 kg/m^2^, and obese: ≥30 kg/m^2^), waist circumference, physical activity (sedentary, moderate, vigorous), SBP, DBP, WBC, total cholesterol, HDL, triglycerides, history of diabetes, hypertension, cardiovascular diseases, cancer, and depression, periodontal diseases, and annual dentist visits. The detailed collection methods for these covariates were similar to the training dataset.

### Cognition assessment

#### Training dataset

Cognition was measured using a version of the Mini-Mental State Examination (MMSE), which was administered through a home interview and at the Mobile Examination Center (MEC) [14–16]. It consisted of six orientation, six recall, and five attention related questions. Each correct response was assigned 1 point and an incorrect response received a score of 0. The outcome used in these analyses was the total score, which was obtained by summing the points assigned to the responses and ranged from 0 to 17 with higher scores indicating better cognitive function. The six orientation items asked about the day of the week, the date, and participant’s complete address including street, city or town, state, and ZIP code (adult questionnaire). To evaluate recall, the interviewer told the participant the names of three items (apple, table, and penny) and asked them to repeat the names. Each participant was given up to six tries to learn the words. If they correctly recalled the items at any of the six tries, the response was considered to be correct. This exercise was repeated after assessing attention to achieve a maximum of 6 points for recall. To evaluate attention, the interviewer asked the participant to serially subtract 3 from 20 and repeat this exercise for up to five times. For example, they were asked to subtract $3 from $20, and $3 from $17, and so on and assigned one point for each correct answer. Based on the prevalence of cognitive impairment among US older adults [17], we defined cognitive impairment as the lowest 10% of the distribution (total score = 10) of the MMSE score.

#### Validation dataset

Cognition was measured using three tests consisting of the Consortium to Establish a Registry for Alzheimer’s Disease (CERAD), the Animal Fluency (AF) test, and the Digit Symbol Substitution Test (DSST) [18]. The CERAD model assesses both immediate and delayed memory of new verbal information [19], the AF test assesses the verbal fluency domain of executive function [20], and DSST assesses attention and processing speed. A score, which is the sum of the total number of correct symbols and number pairs within the time frame was assigned. The assessment was done at the MEC. Individuals in the lowest 10% of the score distribution were categorized as cognitively impaired. Details are provided in **S1 Appendix**. We also used the percentile 20% in the sensitivity analyses.

### Statistical analysis

We developed models to predict cognitive impairment in NHANES III data (training dataset) using logistic regression and machine learning (support vector machine). We used machine learning methods to supplement the logistic regression models because they do not depend on the same modeling assumption. Comparing the results from the machine learning would increase our confidence that the final logistic regression models included all the important variables. We further used those models to predict cognitive impairment in the NHANES 2011 through 2014 (validation dataset). We chose the validation dataset because it had most of the variables that were measured in the training dataset.

#### Training logistic regression model

The binary outcome for cognitive impairment was related to the predictors (cognition-related risk factors) in forward, backward, and stepwise models, from which the most predictive model was selected. We derived the beta coefficients for each variable from the most predictive model to construct the impaired cognition score.

#### Training support-vector machine (SVM) learning models

**Fig 1:**
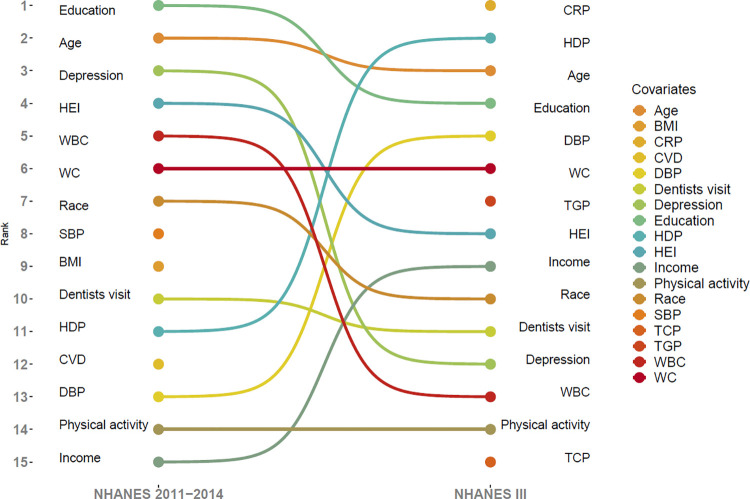
Top 15 covariates for ranked contributions of covariates with poor cognition performance from support vector machine methods in both the development and validation datasets; BMI, body mass index; CRP, C-reactive protein; CVD, cardiovascular diseases; DBP, diastolic blood pressure; HDP, HDL cholesterol; HEI, healthy eating index; SBP, systolic blood pressure; TCP, total cholesterol; TGP, triglycerides; WBC, white blood cell; WC, waist circumference.

We next obtained predictive models in the developmental dataset by relating the outcome (cognitive impairment) with the predictor variables using SVM. We used the 5-fold cross validation method with 30 replications to get the best estimation of the model. We ranked the contribution of each feature and compared the top 15 features from the SVM based on the development and validation datasets (Fig 1). Finally, we constructed the impaired cognition score based on the logistic model using the following formula:

Impairedcognitionscore=0.057×Age−0.017×Waistcircumference−0.012×HEI+0.660×Otherraces+0.416×Blackrace−0.710×Highereducation−0.875×Higherincome−0.325×Middleincome−0.172×Moderatelyactive+0.197×Vigorouslyactive+0.324×Diabetes−0.422×Hypercholesterolemia−0.542×Annualvisittodentist

#### Model validation

To evaluate the performance of the prediction models obtained in the training dataset to external data, we used the final prediction model (logistic model) obtained from NHANES III data to predict impaired cognition in the continuous NHANES 2011 through 2014 data. To do that we calculated a impaired cognition score for each participant using the final logistic regression models obtained earlier and categorized the impaired cognition score into tertiles (low risk, moderate risk, and high risk). We estimated the odds ratios (OR) and the corresponding 95% confidence intervals (CI) using logistic regression to evaluate the association between the impaired cognition score in tertiles and the outcome (impaired cognition) adjusting for age, sex, and race (**Table 3**). We then calculated the area under the curve (AUC) graphs to assess the prediction performance (**Fig 1**).

Data management and statistical analyses were conducted using SAS 9.4 (SAS Institute, Cary, NC). The SVM model was performed in R program (Version 3.5.0, R core team, Vienna, Austria) with the “e1071” package. The sample weights provided by NHANES III were used in all procedures. The threshold for statistical significance was 0.05. The manuscript is compliant with the STROBE statement. The University of South Carolina IRB determined the analyses to be not Human Subjects research.

### Ethical considerations

The NHANES study was approved by National Center for Health Statistics (NCHS) Ethics Review Board (Protocol #2011–17) and documented consent was obtained from all participants.

## Results

### Study population characteristics

We documented 411 participants with low cognitive performance. **Table 1** shows the characteristics of participants in the training dataset from NHANES III. Participants with low cognitive performance compared with their peers with high cognitive performance were older (p<0.001), more likely to be African American, had higher systolic blood pressure (p = 0.001), lower HEI scores (p<0.001), fewer social connection by phone (p<0.001); these individuals were less likely to have visited a dentist in the past year (p<0.001) and more likely to have periodontal disease (p = 0.01).

**Table 1:** Characteristics of the training dataset using participants from NHANES III.

Variable	Normal cognitive performance	Low cognitive performance *	P value
N	3663	411	
Age, mean, year	71.1	75.0	< 0.001
Body mass index, mean, kg/m^2^	27.2	26.1	< 0.001
Waist circumference, mean, cm	97.6	95.5	0.002
Healthy eating index, mean	67.0	61.6	< 0.001
Systolic blood pressure, mean, mmHg	140.7	144.0	0.001
Diastolic blood pressure, mean, mmHg	74.5	73.5	0.07
White blood cells, mean, ×10^9^/L	7.2	7.0	0.10
C-reactive protein, mean, mg/dL	0.6	0.6	0.69
Total cholesterol, mean, mg/dL	222.5	219.3	0.17
HDL cholesterol, mean, mg/dL	51.2	52.3	0.19
Triglycerides, mean, mg/dL	164.1	153.8	0.07
Social connection by phone, mean	10.7	7.7	< 0.001
Social connection with friends/relatives, mean	125.2	120.1	0.57
Social connection with neighbors, mean	83.2	97.1	0.09
Social connection attending church activities, mean	49.1	34.3	0.09
Social connection of meetings in club, mean	13.6	7.7	0.006
Age group, %			< 0.001
40-<65	25.5	13.4	
≥65	74.5	86.6	
Race, %			< 0.001
Whites	80.2	69.6	
Black	17.9	27.5	
Others	1.9	2.9	
Sex, %			0.92
Male	47.2	47.4	
Female	52.8	52.6	
Education, %			< 0.001
<12 years	51.5	80.0	
≥12 years	48.5	20.0	
Income, %			< 0.001
PIR≤1.3	25.3	44.0	
1.3<PIR≤3.5	54.0	50.4	
PIR>3.5	20.7	5.6	
Smoking status, %			0.06
Never smoker	47.2	50.9	
Ever smoker	37.6	31.9	
Current smoker	15.2	17.3	
Ever drinkers, %	34.5	26.0	< 0.001
Body mass index, %			< 0.001
Normal weight	33.9	45.5	
Overweight	41.3	34.5	
Obesity	24.8	20.0	
Physical activity, %			< 0.001
Sedentary	33.8	28.5	
Moderately active	41.0	28.7	
Vigorously active	25.2	42.8	
History of diabetes, yes, %	15.2	20.0	0.01
History of cardiovascular diseases, yes, %	15.8	20.4	0.01
History of hypertension, yes, %	48.2	46.0	0.39
History of cancer, yes, %	18.2	14.6	0.07
History of hyperlipidemia, yes, %	29.6	16.5	< 0.001
Cholesterol-lowering drugs, yes, %	5.6	2.9	0.02
Anxiety, yes, %	0.7	0.7	0.85
Depression, yes, %	3.5	4.4	0.39
Annual visit of dentists, yes, %	36.0	13.6	< 0.001
Periodontal disease, yes, %	49.0	42.8	0.01
Social connection with clubs, yes, %	38.2	23.1	< 0.001

SD, standard deviation; PIR, poverty income ratio.

* Poor cognition performance was defined as the lowest 10% of the distribution of the MMSE score.

In the validation dataset (NHANES 2011–2014), participants with low cognitive performance compared with their peers with good cognitive performance were older (p<0.001), more likely to be African American (<0.001), have higher systolic blood pressure (p<0.001), lower HEI score (p = 0.001), and visited the dentist less frequently in the last year (p<0.001) (**S1 Table in S1 Appendix**).

### Associations between predictors and cognition performance

**Table 2** describes associations between risk factors and cognition performance in the training dataset after adjusting for all covariates. The odds of low cognitive performance were greater with higher age but lower healthy eating index score, fewer social connections by phone, and fewer social connections in attending church activities. Among the participants of the training dataset, the odds of low cognition performance 1.30 times higher for every 5-year increase in age (aOR = 1.30, 95% CI = 1.19–1.41), and 1.58 times higher for African Americans compared with their White peers (aOR = 1.58, 95% CI = 1.15–2.17). The odds of low cognitive performance were 6% lower for a 5-unit increase in the healthy eating index score (aOR = 0.94, 95% CI = 0.89–0.98); 2% lower with more frequent daily social connection by phone (aOR = 0.98, 95% CI = 0.97–0.99); 13% lower with more frequent weekly participation in church activities (aOR = 0.87, 95% CI = 0.76–0.99), and 41% lower among those who visited a dentist in the last year.

**Table 2:** Associations between risk factors and cognition performance in the training dataset from the NHANES III.

Variable	Low cognitive performance *	P value
Age, per 5 years	1.30 (1.19–1.41)	< 0.001
Body mass index, per 5 unit	0.95 (0.74–1.23)	0.70
Waist circumference, per 5 cm	0.94 (0.85–1.05)	0.28
Healthy eating index, per 5 unit	0.94 (0.89–0.98)	0.005
Systolic blood pressure, per SD	1.03 (0.97–1.10)	0.34
Diastolic blood pressure, per SD	0.94 (0.82–1.06)	0.31
White blood cells, per SD	0.93 (0.80–1.08)	0.33
C-reactive protein, per SD	0.90 (0.78–1.03)	0.14
Total cholesterol, per SD	0.99 (0.87–1.14)	0.92
HDL cholesterol, per SD	1.01 (0.87–1.17)	0.91
Triglycerides, per SD	1.02 (0.88–1.18)	0.84
Social connection by phone, daily	0.98 (0.97–0.99)	< 0.001
Social connection with friends/relatives, weekly	0.99 (0.95–1.04)	0.80
Social connection with neighbors, weekly	1.02 (0.98–1.06)	0.45
Social connection attending church activities, weekly	0.87 (0.76–0.99)	0.03
Social connection of meetings in club, weekly	1.04 (0.90–1.21)	0.59
Race		
Whites	1.00 (reference)	
Black	1.58 (1.15–2.17)	0.005
Others	1.92 (0.95–3.85)	0.07
Sex		
Male	1.00 (reference)	
Female	1.04 (0.75–1.44)	0.82
Education		
<12 years	1.00 (reference)	
≥12 years	0.50 (0.37–0.67)	< 0.001
Income		
PIR≤1.3	1.00 (reference)	
1.3<PIR≤3.5	0.73 (0.57–0.95)	0.02
PIR>3.5	0.44 (0.26–0.73)	0.002
Smoking status		
Never smoker	1.00 (reference)	
Ever smoker	0.82 (0.61–1.10)	0.19
Current smoker	0.81 (0.54–1.20)	0.29
Drinking status		
Never drinker	1.00 (reference)	
Ever drinker	0.93 (0.69–1.24)	0.61
Physical activity		
Sedentary	1.00 (reference)	
Moderately	0.86 (0.63–1.16)	0.31
Vigorously	1.22 (0.90–1.66)	0.20
History of diabetes		
No	1.00 (reference)	
Yes	1.32 (0.96–1.83)	0.09
History of cardiovascular diseases		
No	1.00 (reference)	
Yes	1.20 (0.87–1.65)	0.26
History of hypertension		
No	1.00 (reference)	
Yes	0.82 (0.63–1.07)	0.14
History of cancer		
No	1.00 (reference)	
Yes	0.85 (0.60–1.21)	0.37
History of hypercholesterolemia		
No	1.00 (reference)	
Yes	0.64 (0.46–0.90)	0.01
Cholesterol-lowering drugs		
No	1.00 (reference)	
Yes	0.96 (0.48–1.95)	0.92
Anxiety		
No	1.00 (reference)	
Yes	0.88 (0.20–3.96)	0.87
Depression		
No	1.00 (reference)	
Yes	1.48 (0.81–2.68)	0.20
Annual visit of dentists		
No	1.00 (reference)	
Yes	0.59 (0.41–0.83)	0.003
Periodontal diseases		
None	1.00 (reference)	
Moderate	0.99 (0.77–1.27)	0.96

SD, standard deviation; PIR, poverty income ratio.

* Poor cognition performance was defined as the lowest 10% of the distribution of the MMSE score. Data were shown as odds ratios (95% confidence intervals).

### Construction and prediction of the overall impaired cognition score

The top 15 contributors to poor cognitive performance in both the training dataset and validation datasets are shown in **Fig 1**. The highest contributor in the validation dataset was education. In the training dataset, CRP was the highest contributor. Waist circumference and physical activity had the same rank in both datasets. Age was part of the top three contributors in either dataset. Visiting the dentist was ranked tenth in the validation dataset and eleventh in the training dataset, while healthy eating index and race were among the top ten contributors in both the training and validation datasets.

**Table 3** describes the impaired cognition scores for cognitive performance in the training and the validation dataset using the logistic and SVM models. In both datasets, there were higher odds of poor cognition for those in tertiles 2 and 3 when compared to tertile 1. These findings were similar in both the logistic and SVM models in both datasets. In the training dataset, the odds of poor cognition among participants in tertile 3 was 8.15 times the odds among participants in tertile 1 (aOR = 8.15, 95% CI = 5.36–12.4) and 8.77 times that of the odds among participants in tertile 1 (aOR = 8.77, 95% CI = 5.57–13.8) for the logistic and SVM models respectively. In the validation dataset, the odds of poor cognition among those in tertile 3 was 4.31 times the odds of participants in tertile 1 (aOR = 4.31, 95% CI = 2.62–7.08) and 3.95 times that of the odds among participants in tertile 1 (aOR = 3.95, 95% CI = 2.34–6.66) for the logistic and SVM models respectively. Using 20% as the cutoff of impaired cognitive performance in the validation dataset yielded similar results (**S2 Table in S1 Appendix**).

**Table 3:** Impaired cognition score for cognition performance based on the development and prediction datasets.

	Tertiles of impaired cognition scores *		
	Tertile 1	Tertile 2	Tertile 3
**NHANES III (training dataset)**			
Impaired cognition score based on logistic model			
Cases/Controls †	27/1186	91/1123	217/996
Model 1, OR (95% CI) ‡	1.00 (reference)	3.56 (2.30–5.51)	9.57 (6.36–14.4)
Model 2, OR (95% CI) §	1.00 (reference)	3.18 (2.05–4.94)	8.15 (5.36–12.4)
Impaired cognition score based on SVM model			
Cases/Controls	25/1188	82/1132	228/985
Model 1, OR (95% CI)	1.00 (reference)	3.44 (2.18–5.43)	11.0 (7.22–16.8)
Model 2, OR (95% CI)	1.00 (reference)	3.18 (2.01–5.04)	8.77 (5.57–13.8)
**NHANES 2011–2014 (validation dataset)**			
Impaired cognition score based on logistic model			
Cases/Controls	25/811	47/790	113/724
Model 1, OR (95% CI)	1.00 (reference)	1.93 (1.18–3.17)	5.06 (3.25–7.90)
Model 2, OR (95% CI)	1.00 (reference)	1.66 (0.99–2.76)	4.31 (2.62–7.08)
Impaired cognition score based on SVM model			
Cases/Controls	21/815	44/793	120/717
Model 1, OR (95% CI)	1.00 (reference)	2.15 (1.27–3.65)	6.50 (4.04–10.4)
Model 2, OR (95% CI)	1.00 (reference)	1.59 (0.92–2.74)	3.95 (2.34–6.66)

NHANES, National Health and Nutrition Examination Survey; SVM, support vector machine; OR, odds ratio; CI, confidence interval.

* The tertiles was based on the distribution of all participants.

† Poor cognition performance was defined as the lowest 10% of the distribution of the MMSE score (NHANES III) or a composite score based on the Consortium to Establish a Registry for Alzheimer’s Disease (CERAD), the Animal Fluency (AF), and the Digit Symbol Substitution Test (DSST) tests (NHANES 2011–2014).

‡ Model 1 is the crude model without any adjustments.

§ Model 2 adjusted for age, sex, and race.

**Fig 2:**
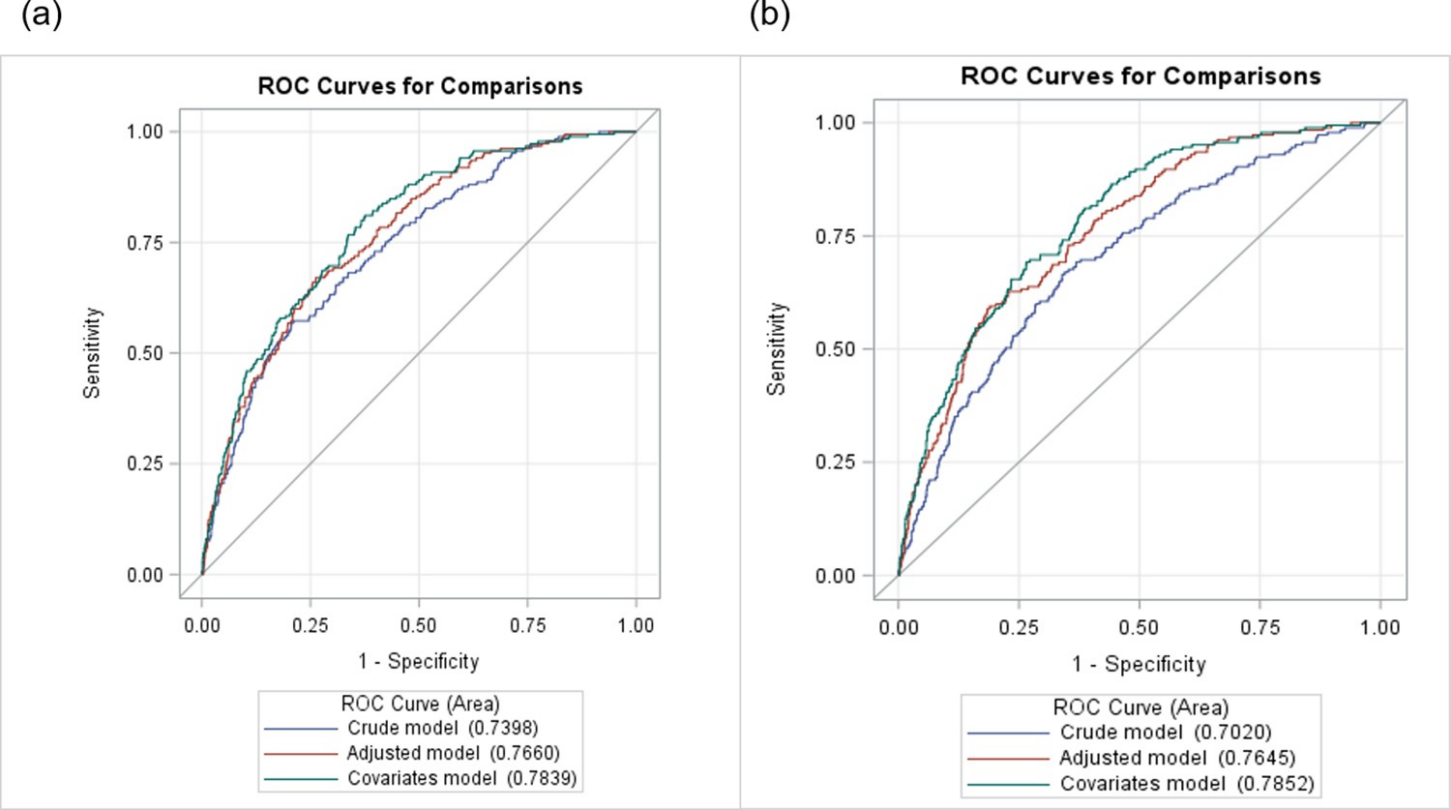
Performance of the prediction model using both logistic regression and support vector machine methods in the validation datasets; (a) logistic model; (b) SVM model. Adjusted model included age, sex, and race. Covariates model additionally adjusted for education, history of cardiovascular disease, depression, and white blood cells.

The logistic and SVM models had similar discriminatory abilities in predicting poor cognition using the validation dataset. The area under the curve (AUC) for the crude model in was 0.74 and 0.70 for the logistic model and SVM model, respectively. The AUC for the age, sex and race-adjusted model in the logistic and SVM models was 0.77 and 0.76 respectively, and the AUC for the models that adjusted for all covariates in the logistic and SVM methods was 0.78 and 0.79 respectively (**Fig 2**).

## Discussion

We developed a model using statistical and machine learning methods that predicted impaired cognition using ten known risk factors and validated it in a replication dataset among free-living older adults. Information on the risk factors in our model is commonly available in electronic health records but is not systematically used to predict impaired cognition. The model could be embedded in an electronic health record system to predict the likelihood of impaired cognition for individuals using information in their respective health records, which could assist their health care providers to decide on the next steps. The positive predictive value would be increased for all tools used to evaluate impaired cognition by screening individuals identified as high-risk using our model, thus facilitating the diagnosis of impaired cognition among older adults.

Although the causes of cognitive impairment are not yet completely understood, evidence of associations between individual risk factors and cognitive loss is accumulating. Besides increasing age, low education level is associated with higher risk of cognition impairment [21], Some unhealthy lifestyles, including smoking, lack of physical activity, unhealthy eating are potential risk factors [22]. In addition, people with diabetes, obesity, depression, hypertension, and hyperlipidemia have an increased risk of developing cognition decline [23]. However, few studies focused on a combination of these risk factors. Kivipelto and colleagues developed the CAIDE impaired cognition score based on modifiable risk factors that predicted the incidence of dementia over 20 years [24]. Combinations of risk factors have been shown to predict dementia incidence over time in other prospective studies [5, 25–27]. In contrast, we combined information on risk factors to predict the prevalence of impaired cognition. Our goal was to facilitate case identification at one moment in time rather than predicting dementia risk over time. Tolea and colleagues using a modified CAIDE instrument in a cross-sectional analysis reported an AUC of 0.63 to predict mild cognitive impairment versus no impairment [28]. In comparison, AUC estimates to assess impaired cognition in our study ranged from 0.70 to 0.79 in various models.

The USPTF does not recommend routine screening for cognitive impairment in older adults (≥65 years) [29] due to the insignificant clinical benefit. Screening tools are effective in identifying cognitive impairment, do not cause any harm, and but benefits from subsequent interventions are modest [30]. Early diagnosis of impaired cognition may nevertheless benefit individuals and their caregivers to manage and monitor the condition and plan for the future, and health care providers to focus on reversing modifiable risk factors and making appropriate treatment plans. Among nationally representative older US adults, 12 potentially modifiable risk factors accounted for 41% of dementia cases overall and 46% and 47% of dementia cases among African Americans and Hispanics respectively [31]. Another analysis of a nationally representative sample of US adults identified 8 modifiable risk factors that contributed to 35.9% and 30.1% of ADRD cases in men and women respectively, 40% of cases among African Americans, and 34% of cases among Hispanics [32]. Additionally, it has been shown that pathologic changes in the brain may precede development of symptoms by several years [33]. Together these studies suggest that managing risk factors early, particularly in high-risk groups, may be beneficial in reducing disease burden. Our model identifies individuals likely to have impaired cognition which could help health care providers to diagnose impaired cognition.

Our study has several strengths. We applied different methods (traditional logistic regression and SVM models) to derive robust and stable models. Though a systematic review found no benefit in machine learning over logistic regression in clinical prediction [34], machine learning may perform better than logistic regression in smaller samples [35], and with high dimension data [36]. The reason for using both logistic regression and SVM models was to increase the chances that important variables would be included in the final prediction model. Though we used logistic regression as the final predictive model, at the outset we did not know which variables would be selected by which method. Using both methods to identify risk factor clusters may therefore have been an advantage. Additionally, the training and validation datasets were from samples representative of free-living older US adults. However, our study also has some limitations. For example, the predictive properties of our model are in the acceptable range but are not excellent [37]. One possible reason for this is that the NHANES datasets were not specifically designed for evaluating cognition. The model could be refined in a dataset with more accurate measures of cognition such as more extensive neurocognitive testing, and neuroimaging such as MRI and EEG [38]. Another way to strengthen the model is by evaluating a wider range of risk factors such as sleep, physical function, speech and hearing abilities, vision, reading history, access to healthcare, social interactions, family engagement, and oral health [38–42]. Many of these factors have been related with cognition but are not formally used to predict undiagnosed cognitive impairment. For example, though poor oral health is associated with worse cognition, oral health information is not widely used to predict impaired cognition. Another limitation is that the statistical approach used to identify risk factors assumes that the model assumptions are met. For example, to identify the initial set of possible predictors we used logistic regression, which assumes that the relation between continuous risk factors and the log odds of the outcome are linearly related. A violation of this assumption could result in loss of efficiency. To address this concern, we repeated the variable selection process using machine learning methods which do not have this limitation. The final list of predictors was obtained using information from both logistic regression and machine learning methods. Another limitation was that some domains were measured differently in the development (NHANES III) and validation datasets (continuous NHANES 2011–2014). For example, the cognitive score assessed in the validation dataset used newer methods. Though we used the same overall criteria to evaluate cognitive impairment (lowest 10% of cognition score) in both the development and validation datasets, cognitive assessment in the latter dataset was likely more accurate. This could also have reduced the predictive properties of the model. Despite these limitations, the approach seems promising for clinical translation. For example, based on the predicted probability of cognitive impairment obtained for a particular patient from the model, a health care provider could decide whether to test that patient.

## Conclusions

We developed a model consisting of 10 common risk factors that predicted undiagnosed cognitive impairment in a representative sample of older US adults. This model could be used to identify groups with greater prevalence of cognitive impairment in its current form to aid in screening. This model could be enhanced, and its prediction improved by refining it in a richer dataset with more accurate and varied outcome measures and a broader range of predictors.

## Supporting information

S1 Appendix (DOCX)

## Abbreviations

AD: Alzheimer’s Disease
AF: the Animal Fluency test
AUC: area under the curve
BMI: body mass index
CERAD: the Consortium to Establish a Registry for Alzheimer’s Disease
CI: confidence intervals
CRP: C-reactive protein
DBP: diastolic blood pressure
DSST: the Digit Symbol Substitution Test
HDL: high density lipoprotein cholesterol
HEI: the healthy eating index
NHANES: the Third National Health and Nutrition Examination Survey
OR: odds ratios
SBP: systolic blood pressure
WBC: white blood cell count
