# Supplementary material for: Identifying a group of factors predicting cognitive impairment among older adults
Source: PLoS One. 2024 Apr 11;19(4):e0301979. doi: 10.1371/journal.pone.0301979 (PMC11008866; doi:10.1371/journal.pone.0301979)
Supplement: S1 Appendix — (DOCX) [file pone.0301979.s001.docx]

**Supplementary Appendix**

This appendix has been provided by the authors to give readers additional information about their work.

Supplement to: Zhao L, Wang Y, Mishio Bawa E, et al. Identifying a group of factors predicting cognitive impairment among older adults

Supplemental materials

**Appendix: Supplemental methods**

**Cognition (outcome): Validation dataset**

Cognition was measured using word list learning trials with a delayed recall from the Consortium to Establish a Registry for Alzheimer’s Disease (CERAD), the Animal Fluency (AF) test, and the Digit Symbol Substitution Test (DSST). The CERAD model assesses both immediate and delayed memory of new verbal information (Moms et al., 1989; Morris et al., 1989). It was made up of three consecutive word learning trials of 10 words. The participants read the words aloud from a computer screen, followed by an immediate recall. The 10-item word list (CERAD-WL) was the same for each trial. The score was an average of the number of recalled words across the consecutive trials The delayed recall (CERAD-DR) of all 10 words was carried out after the AF and DSST assessment without a review of the word list. Participants were asked to recall as many words are possible from the 10 words presented originally. To examine verbal semantic fluency, the AF test was used. The AF test assesses the verbal fluency domain of executive function (Strauss et al., 2006). Participants were made to name as many animals as possible in 1 minute aloud. They were first made to name three articles of clothing as a pretest. Those who could not correctly name articles of clothing did not proceed with the AF test. One point was awarded for each animal named. The DSST was used to evaluate attention and processing speed. It is a subset of the Wechsler Adult Intelligence Scale, Third Edition (WAIS-III) (Boake, 2002). With a key/legend located at the top of the paper test, 9 numbers are paired with unique symbols. Rows of 133 adjoining boxes with a number in the top portion of each box were beneath the key/legend. Participants had 2 minutes to copy the symbol that matches the number below each box using the key. Before the DSST, participants were shown how to perform the task and asked to practice. Those who were unable to complete the short practice exercise did not take part in the full DSST. A score, which is the sum of the total number of correct symbols and number pairs within the time frame was assigned. The assessment was in the order: CERAD-WL, AF, DSST, CERAD-DR. This order was fixed except that, only DSST, a paper and pencil instrument, was administered when participants refused to consent to a recording. The assessment was done in the Mobile Examination Center (MEC). Individuals in the lowest 10% of the score distribution were categorized as cognitively impaired.

**Supplemental Table 1.** Characteristics of the training dataset using participants from NHANES 2011-2014

| Variable | Normal cognitive performance | Low cognitive performance | P value |
| --- | --- | --- | --- |
| N | 2325 | 185 |  |
| Age, mean, years | 69.1 | 72.7 | < 0.001 |
| Body mass index, mean, kg/m^2^ | 29.1 | 28.2 | 0.05 |
| Waist circumference, mean, cm | 102.1 | 101.8 | 0.8 |
| Healthy eating index, mean | 58.0 | 54.7 | 0.001 |
| Systolic blood pressure, mean, mmHg | 132.6 | 138.7 | < 0.001 |
| Diastolic blood pressure, mean, mmHg | 67.9 | 67.1 | 0.46 |
| White blood cells, mean, ×10^9^/L | 6.9 | 7.5 | 0.001 |
| Total cholesterol, mean, mg/dL | 192.4 | 183.3 | 0.005 |
| HDL cholesterol, mean, mg/dL | 54.6 | 53.2 | 0.26 |
| Age group, % |  |  | < 0.001 |
| 40-<65 | 56.3 | 33.0 |  |
| ≥65 | 43.7 | 67.0 |  |
| Race, % |  |  | < 0.001 |
| Whites | 49.7 | 34.6 |  |
| Black | 22.0 | 32.4 |  |
| Others | 28.3 | 33.0 |  |
| Sex, % |  |  | 0.01 |
| Male | 49.0 | 58.4 |  |
| Female | 51.0 | 41.6 |  |
| Education, % |  |  | < 0.001 |
| <12 years | 22.8 | 53.0 |  |
| ≥12 years | 77.2 | 47.0 |  |
| Income, % |  |  | < 0.001 |
| PIR≤1.3 | 25.2 | 43.2 |  |
| 1.3<PIR≤3.5 | 43.3 | 40.5 |  |
| PIR>3.5 | 31.4 | 16.2 |  |
| Smoking status, % |  |  | 0.27 |
| Never smoker | 49.8 | 46.5 |  |
| Ever smoker | 36.6 | 35.7 |  |
| Current smoker | 13.6 | 17.8 |  |
| Ever drinkers, % | 68.9 | 67.6 | 0.71 |
| Body mass index, % |  |  | 0.70 |
| Normal weight | 26.0 | 27.0 |  |
| Overweight | 35.8 | 37.8 |  |
| Obesity | 38.2 | 35.1 |  |
| Physical activity, % |  |  | < 0.001 |
| Sedentary | 44.3 | 61.6 |  |
| Moderately active | 34.6 | 25.4 |  |
| Vigorously active | 21.1 | 13.0 |  |
| History of diabetes, yes, % | 22.3 | 32.4 | 0.001 |
| History of cardiovascular diseases, yes, % | 20.5 | 34.6 | < 0.001 |
| History of hypertension, yes, % | 61.9 | 65.9 | 0.27 |
| History of cancer, yes, % | 20.6 | 13.5 | 0.02 |
| History of hyperlipidemia, yes, % | 56.7 | 56.2 | 0.89 |
| Depression, yes, % | 8.2 | 16.2 | < 0.001 |
| Annual visit of dentists, yes, % | 62.4 | 40.0 | < 0.001 |
| Periodontal disease, yes, % | 17.1 | 13.0 | 0.15 |

HDL, high-density lipoprotein cholesterol; NHANES, National Health and Nutrition Survey; PIR, poverty income ratio.

Poor cognition performance was defined as the lowest 10% of the distribution of a composite score based on the Consortium to Establish a Registry for Alzheimer’s Disease (CERAD) model, the Animal Fluency (AF) test, and the Digit Symbol Substitution Test (DSST).

**Supplemental Table 2.** Impaired cognition score for cognition performance based on the prediction datasets (NHANES 2011-2014)

| **Using percentile 20% as cutoff of impaired cognition** | **Tertiles of impaired cognition scores** * | | |
| --- | --- | --- | --- |
|  | **Tertile 1** | **Tertile 2** | **Tertile 3** |
| Impaired cognition score based on logistic model | | |  |
| Cases/Controls † | 54/782 | 115/722 | 244/593 |
| Model 1, OR (95% CI) ‡ | 1.00 (reference) | 2.31 (1.64-3.24) | 5.96 (4.36-8.15) |
| Model 2, OR (95% CI) § | 1.00 (reference) | 2.00 (1.41-2.85) | 5.28 (3.71-7.52) |
| Impaired cognition score based on SVM model | |  |  |
| Cases/Controls | 47/789 | 116/721 | 250/587 |
| Model 1, OR (95% CI) | 1.00 (reference) | 2.70 (1.90-3.85) | 7.15 (5.14-9.94) |
| Model 2, OR (95% CI) | 1.00 (reference) | 2.06 (1.43-2.98) | 4.60 (3.19-6.62) |
| NHANES, National Health and Nutrition Examination Survey; SVM, support vector machine; OR, odds ratio; CI, confidence interval.  * The tertiles was based on the distribution of all participants. † Poor cognition performance was defined as the lowest 10% of the distribution of the MMSE score (NHANES III) or a composite score based on the Consortium to Establish a Registry for Alzheimer’s Disease (CERAD), the Animal Fluency (AF), and the Digit Symbol Substitution Test (DSST) tests (NHANES 2011-2014).  ‡ Model 1 is the crude model without any adjustments.  § Model 2 adjusted for age, sex, and race. | | | |

**Supplemental Figure 1.** Flowchart of study population

Total participants in the NHANES 2011-2012 and 2013-2014 (n=19,931)

Total participants in the NHANES III (n=7869)

Excluded due to:

1. No valid cognition measurement (n=16,459)

2. Missing values in the covariates (n=962)

Final validation dataset (n=2510)

Excluded due to missing values in the covariates or cognition variables (n=3795)

Final training dataset (n=4074)
